# Maryland & D. C. Dental Association Proceedings

**Published:** 1879-06

**Authors:** 


*Proceedings of the Dental Association of the State of Maryland and the District of Columbia*, Washington, October 8th, 9th and 10th, 1878.

A volume of 96 pages, just received. An abstract of these proceedings was given in the November number of this Journal, and several of the papers published in part. Most of the papers presented are well worthy of a careful perusal. It is to be wished that greater promptness were exercised in bringing out such proceedings. Seven months is certainly a needless delay.

The paper of Dr. B. F. Coy, in these Transactions, on the Treatment of the Six Year Molars, contains some points of interest, as the proper time for the extraction of teeth to relieve the overcrowded jaw is a question of great importance. Dr. Coy fixes on eleven years as the most desirable time for the removal of the first permanent molars, where their removal is indicated. He concludes "that in nineteen cases out of twenty it (the extraction of these teeth) is beyond doubt the true course to pursue at the present day."

In the discussion which took place on the question of transmitted tendencies, Prof. Winder took the ground that, contrary to the teaching of the books, eight children out of ten take their teeth from the father, rather than from the mother.

The paper of Dr. J. J. Caldwell, on Anæsthesia and Anæsthetics, is an exceedingly full digest of the status of the profession on these subjects, and is the most voluminous of the papers presented. He quotes largely from the standard authorities, and the abstracts from Dr. Dabney, of Virginia, especially, are full of interest.

A remark of Dr. Coy on the subject of the administration of anæsthetics is worthy of quotation: "I am a firm believer in having the surroundings quiet during the administration of any anæsthetic, and of permitting the patient to recover if possible, without too much officiousness on the part of the operator or his assistant—relatives and friends scarce, or on the back seats. * * Nervousness or indecision on the part of the operator is a bad preparation for the patient who is to submit to the influence of such powerful agents. * * One great point has been gained when you have secured the full confidence of your patient; another when you are sure you have the requisite confidence in yourself."

The paper of Dr. W. H. Atkinson, of New York, on diagnosis and therapeutics, contains some thoughts of interest, less entangled with mystic verbiage than is usual in the author's style. Still, either he is very far ahead, or we, in company with many others, are very far behind in apprehension of ideas as clothed in language, for much of the language used by him is simply unintelligible—to us.

Dr. Caldwell's papers (this gentleman, although a practising physician, seems so fond of the dental associations, as to be almost regularly present at some of them,) on Diagnosis and Therapeutics, with a review of Alcoholism and Syphilis, might at first sight seem out of place in the transactions of a dental association, but they were *not* out of place, and were very nicely conceived and expressed documents. The opinions of Dr. Caldwell as to the extreme importance of checking by National Legislation the spread of syphilis, should be carefully considered, and his idea as to the deep injury to mankind by the free prescribing of alcoholic stimulants by physicians, is a truism too little pondered on.

Other papers, by Dr. W. W. Evans, on Mechanical Dentistry, and by Dr. H. B. Noble, on Dental Education and Literature, are thoughtful and suggestive essays. It is hoped that in future such publications will be more carefully proof-read. Many errors mar this pamphlet.

The next meeting of this Association will be in Baltimore, when a largely increased interest is expected. H.

